# A risk progression breast epithelial 3D culture model reveals Cx43/hsa_circ_0077755/miR-182 as a biomarker axis for heightened risk of breast cancer initiation

**DOI:** 10.1038/s41598-021-82057-y

**Published:** 2021-01-29

**Authors:** Nataly Naser Al Deen, Nadia Atallah Lanman, Shirisha Chittiboyina, Sophie Lelièvre, Rihab Nasr, Farah Nassar, Heinrich Zu Dohna, Mounir AbouHaidar, Rabih Talhouk

**Affiliations:** 1 Department of Biology, Faculty of Arts and Sciences, American University of Beirut, P.O. Box: 11-0236, Beirut, Lebanon; 2 Purdue University Center for Cancer Research, West Lafayette, IN USA; 3 Department of Comparative Pathobiology, College of Veterinary Medicine, Purdue University, West Lafayette, IN USA; 4 Department of Basic Medical Sciences, College of Veterinary Medicine, Purdue University, West Lafayette, IN USA; 5 Department of Anatomy, Cell Biology and Physiological Sciences, American University of Beirut, Beirut, Lebanon; 6 Department of Internal Medicine, Faculty of Medicine, American University of Beirut, Beirut, Lebanon; 7 Department of Cell and Systems Biology, University of Toronto, 25 Willcocks St., Toronto, ON M5S 3B2 Canada

**Keywords:** Breast cancer, Apicobasal polarity, Mechanisms of disease, Next-generation sequencing

## Abstract

mRNA-circRNA-miRNAs axes have been characterized in breast cancer, but not as risk-assessment axes for tumor initiation in early-onset breast cancer that is increasing drastically worldwide. To address this gap, we performed circular RNA (circRNA) microarrays and microRNA (miRNA) sequencing on acini of HMT-3522 S1 (S1) breast epithelial risk-progression culture model in 3D and chose an early-stage population miRNome for a validation cohort. Nontumorigenic S1 cells form fully polarized epithelium while pretumorigenic counterparts silenced for gap junction Cx43 (Cx43-KO-S1) lose epithelial polarity, multilayer and mimic premalignant in vivo mammary epithelial morphology. Here, 121 circRNAs and 65 miRNAs were significantly dysregulated in response to Cx43 silencing in cultured epithelia and 15 miRNAs from the patient cohort were involved in epithelial polarity disruption. Focusing on the possible sponging activity of the validated circRNAs to their target miRNAs, we found all miRNAs to be highly enriched in cancer-related pathways and cross-compared their dysregulation to actual miRNA datasets from the cultured epithelia and the patient validation cohort. We present the involvement of gap junction in post-transcriptional axes and reveal Cx43/hsa_circ_0077755/miR-182 as a potential biomarker signature axis for heightened-risk of breast cancer initiation, and that its dysregulation patterns might predict prognosis along breast cancer initiation and progression.

## Introduction

Breast cancer accounts for the highest cancer incidence (24.2%) and mortality rate (15%) in women globally and is the second most commonly diagnosed cancer (after lung cancer) when both sexes are combined (11.6%)^1^. In Lebanon, breast cancer constitutes one-third of all female cancers, with an alarming high percentage diagnosed under the age of 40 (22% of cases compared to 6% in Western populations) and a mean age at diagnosis 10 years younger than in Western countries^2^. These women have low prevalence of deleterious *BRCA* mutations^3^ and present with poor prognosis and aggressive phenotypes due to the lack of diagnostic methods at such an early age^4^. Early-onset breast cancer is increasing drastically worldwide^2,5,6^, which called for this study to identify potential noninvasive biomarkers and active players^7,8^ for risk-assessment and early detection actions.

Among key players involved in breast cancer onset are gap junction components. Gap junction intercellular communication (GJIC) is mediated by transmembrane proteins, called connexins (Cxs) that allow the intercellular exchange of ions, second messengers and metabolites between adjacent cells^9–12^. Cx43, the focus of our previous and current research studies^8,13,14^, plays essential roles during mammary gland development^15,16^ and differentiation^17^ and acts as a tumor suppressor^13,14,18^. Its loss and mislocalization influence breast cancer initiation^19^, progression^20^, increase risk of breast cancer development in overweight women^21,22^ and is associated with markers of poor prognosis, increased metastasis and poor survival in breast cancer patients^18^. We recently showed that Cx43 functions via PI3 Kinase and noncanonical Wnt signaling pathways in priming the breast epithelium for neoplastic behavior^19,20^. The nontumorigenic luminal human breast epithelial HMT-3522 S1 (S1) cell line, cultured under three-dimensional (3D) conditions, forms growth-arrested and basoapically polarized acini with a central lumen and apicolateral localization of Cx43. Hence S1 cells recapitulate normal human breast tissue architecture^19^. Silencing Cx43 expression in these nontumorigenic S1 cells via Cx43-shRNA (Cx43-KO-S1) resulted in cell cycle entry, perturbed apical polarity, mitotic spindle misorientation and loss of lumen, causing cell multilayering^19^ and priming cells for enhanced motility and invasion^19,20^. These phenotypic features observed in Cx43-KO-S1 acini represent architectural and phenotypical premalignant mammary lesions, like those observed in ductal hyperplasia in a murine model^23^, which increase the risk of breast cancer initiation, thus marking Cx43-KO-S1 as a pretumorigenic culture model. This 3D risk-progression culture model was therefore utilized to capture key pretumorigenic signatures that might delineate post-transcriptional profiles seen in the early-stage patients with heightened risk of breast cancer development.

Cancerous phenotypes have been shown to be mediated by circRNAs, a class of endogenous RNAs that originate from RNA splicing and back ligation and act as miRNA “sponges”, and miRNAs, small endogenous non-coding RNAs that repress translation^8,24^. CircRNAs are covalently closed continuous loops without 5′ cap or 3′ polyadenylated tail and are resistant to degradation by exonucleases (e.g. RNase R) that degrade linear RNA^25–27^. One of the known functions of circRNAs^28^ includes “sponging” mature miRNAs and RNA-binding proteins (RBP)s. Sponging refers to circRNAs exhibiting endogenous competing binding sites (one to several) for a specific target miRNA. They covalently bind the mature target miRNA, thus downregulating its expression or sequester the miRNA from other target genes, and thereby limiting their activity to repress the translation of its own target mRNAs^29^. For instance, ciRS-7 exhibits over 70 conserved binding sites to miR-7^30^ while circ-SRY exhibits16 binding sites to miR-138^29,31^ and circ-Foxo3^32^ and circ-MBL (muscleblind) can sponge RBPs^33^. Other functions of circRNAs include (i) cell cycle regulation, for example, by interacting with CDK2 and p21, circ-Foxo3 can block cell cycle progression from G1 to S phase^32^, (ii), translation of exonic circRNAs with open reading frames, for example, through an internal ribosome entry site, IRES, hcirc-ZNF609 is capable of translating proteins in mouse myoblasts^34^, (iii) control of stability of some mRNAs, for instance, CDR1as circular antisense RNA can stabilize mRNAs through formation of a duplex structure^26^, (iv) positive regulation and modulation of their own parental gene expression through association with RNA polymerase II (like ci-ankrd52 and ci-sirt7^35^) and (v) regulation of alternative splicing^28,33^.

While dysregulated mRNA-circRNAs-miRNAs axes have been reported as biomarker signatures in cancers^30,36–38^ and specifically in breast cancers^39–45^, axes involved in premalignant epithelial polarity transitions that might explain and contribute to heightened risk of breast cancer initiation have not been reported. We therefore, through utilizing a unique breast cancer risk-progression 3D culture model that mimics normal and premalignant in vivo mammary epithelial morphology^19,46^ and profiles of a validation cohort of early-stage Lebanese patients that is notoriously at highest risk for the early malignancy with 42.2% below the age of 40^2,47^, characterized new potential post-transcriptional risk-assessment axes of breast cancer initiation.

**Figure 1:**
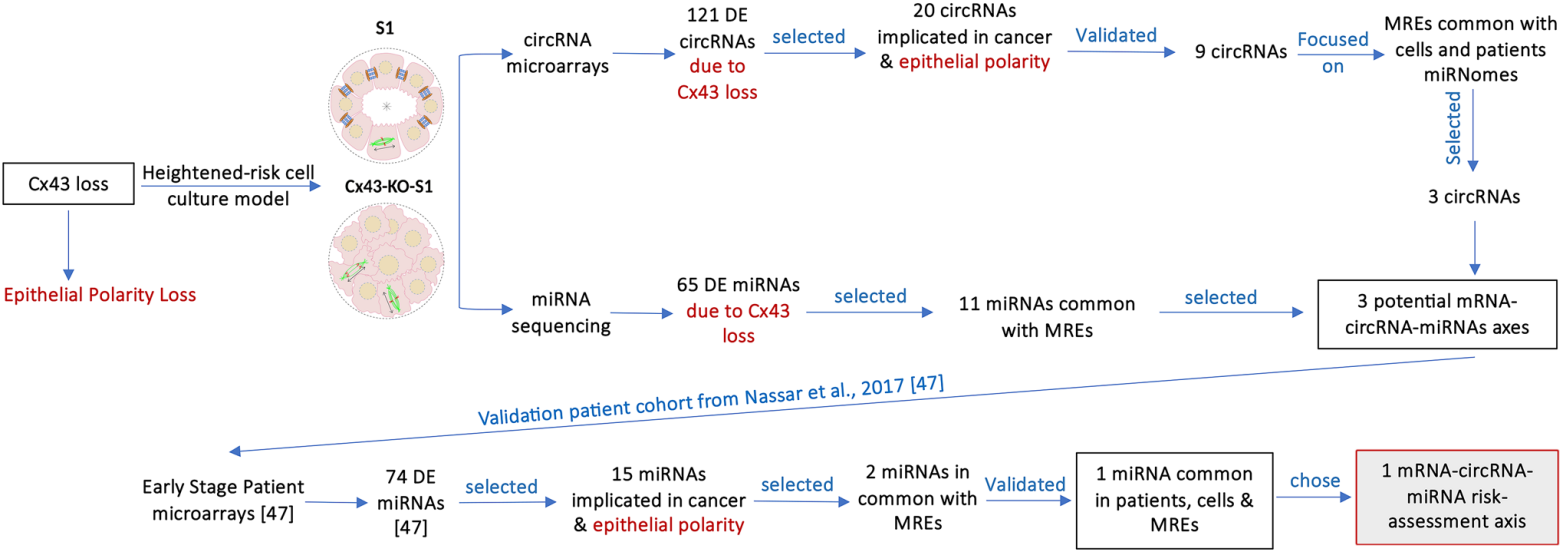
A graphical abstract summarizing the methodology used to select Cx43/has_circ_0077755/miR-182 as the only validated risk-assessment axis for breast cancer initiation. Using the circRNA microarrays and miRNA sequencing results of Cx43-KO-S1 compared to S1 cells and focusing mainly only on Cx43 loss (and hence epithelial polarity loss) and on the sponging activity of circRNAs to miRNAs, three axes were predicted for breast cancer risk-assessment. After using a validation early-stage young breast cancer patient cohort as published in Nassar et al.^47^, the list was narrowed down to only Cx43/has_circ_0077755/miR-182 axis. MREs refer to miRNA response elements, predicted to be “sponged” by the significant circRNAs based on Arraystar's miRNA target prediction software^52,53^.

In recent work^8^, in an attempt to predict such post-transcriptional axes, we predicted in silico potential Cx43 mRNA-circRNAs-miRNAs biomarker signatures for early-onset breast cancer risk assessment, which might recapitulate phenotypes observed upon Cx43 loss^14,37,48,49^. In the present report, dysregulated mRNA-circRNA-miRNAs signature axes were studied using circRNA microarray and miRNA sequencing analysis of nontumorigenic S1 and pretumorigenic Cx43-KO-S1 cells, and microarrays of a validation young patient cohort with early-stage breast cancer^47^ and large cohort datasets of breast cancer miRnome and transcriptome^50,51^. The potential biomarker roles of three validated significantly dysregulated circRNAs were investigated. We focused on their possible sponging capacity to their target miRNAs in the breast cancer risk progression 3D culture model and looked for matched dysregulation in the patient validation cohort. Only Cx43/hsa_circ_0077755/miR-182 axis, specific to Cx43 loss, was validated (Fig. 1), might serve as a biomarker signature axis for heightened-risk of breast cancer initiation and its dysregulation patterns seem to predict prognosis along breast cancer initiation and progression.

## Results

### Three-dimensional (3D) breast cancer risk-progression cell culture model characteristics

S1 nontumorigenic cells^46^ have been used previously in 3D cell culture, in the presence of extracellular matrix of basement membrane type, as a model of phenotypically normal differentiation of breast luminal epithelium^54,55^. They form a fully polarized epithelium that displays apicolateral distribution of tight junction proteins ZO-1 and ZO-2^56,57^. We have shown in previous studies that Cx43 drives apical polarity in vivo, and that it controls the distribution of tight junction proteins and of adherens junction component β-catenin (Supplementary Fig. 1a)^19^. Its loss is associated with cell multilayering and polarity disruption as shown in the S1 epithelium silenced for Cx43 (pretumorigenic Cx43-KO-S1 cells) (Supplementary Fig. 1a,b) and in archival biopsy tissue samples^19^. This in vitro 3D risk-progression culture model was used here to study the post-transcriptional signature axes (namely miRNAs and circRNAs) that are dysregulated as a result of Cx43 loss in the breast epithelium, a feature commonly associated with heightened risk of breast cancer initiation^18,22^.

### Microarray profiling of Cx43-KO-S1 (pretumorigenic) versus S1 (nontumorigenic) breast epithelial cells in 3D culture revealed 121 significantly dysregulated circRNAs, of which 18 were chosen for validation

**Figure 2:**
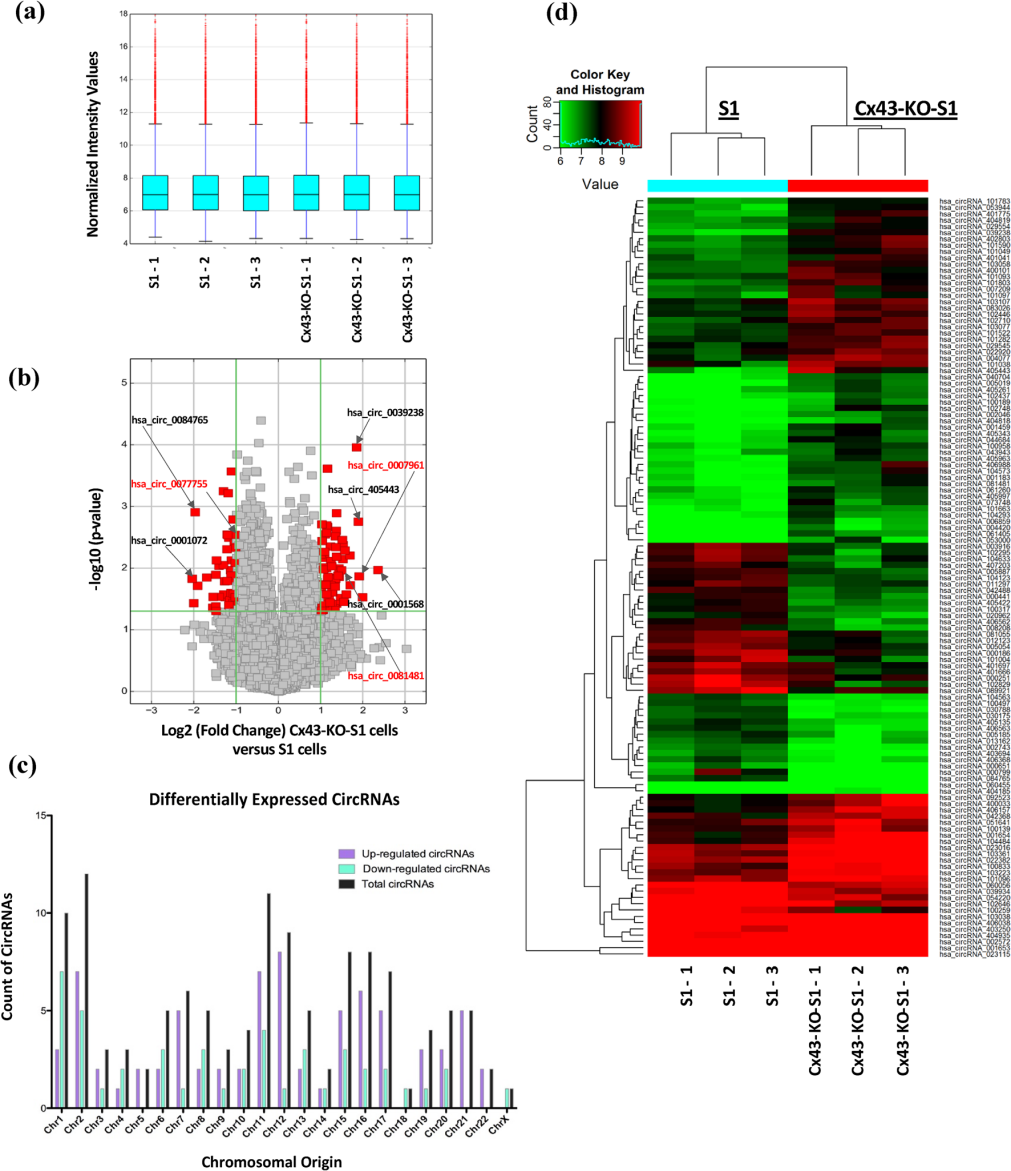
Microarrays revealed 121 differentially expressed circRNAs in response to Cx43 silencing in Cx43-KO-S1 (pretumorigenic) cells versus S1 (nontumorigenic) breast epithelial cells in 3D. Triplicates of Cx43-KO-S1 and triplicates of S1 cells were plated on Matrigel™ for 11 days. Total RNA was extracted, digested with RNase R to remove linear RNAs and enrich circRNAs, reverse transcribed and hybridized to Arraystar Human circRNA Array V2 microarrays. (**a**) Box plot after quantile normalization showing the distributions of log2 ratios among the six samples. (**b**) Volcano plot depicting the differential circRNA expression, with the vertical green lines corresponding to 2.0-fold up and down, and the horizontal green line representing a p-value of 0.05. The red points in the plot represent the differentially expressed circRNAs with statistical significance. The circRNAs denoted in black font with arrows highlight the most up-regulated (right) and down-regulated (left) circRNAs, while the circRNAs denoted in red font with arrows highlight the three chosen and validated circRNAs in this study. (**c**) Bar graph showing the chromosomal distributions of the differentially expressed circRNAs. (**d**) Unsupervised hierarchical cluster analysis (heat map) of microarray data used to assess the significant expression of circRNAs when comparing Cx43-KO-S1 to S1 cells in 3D (the key range (6–10) represents the log2 value of the normalized intensity for each sample and not the fold change). “Red” indicates higher expression level, and “green” indicates lower expression level in Cx43-KO-S1 as compared to S1 cells. Each circRNA is represented by a single row of colored boxes and each sample is represented by a single column.

To identify circRNA expression profile specific to the loss of Cx43, microarrays for circRNAs (Arraystar Human circRNA Array V2) were performed. Triplicates of Cx43-KO-S1 cells and S1 counterparts in 3D drip culture were prepared for the microarray in the presence of Matrigel™ over 11 days to induce the formation of acinus-like structures. CircRNA expression levels were processed and analyzed upon sample quantile normalization (Fig. 2a). Differential expression levels of circRNAs showed that hsa_circ_0001568 (originating from *DUSP22* gene), hsa_circ_405443 (originating from *NDE1* gene) and hsa_circ_0039238 (originating from *NETO2* gene) were the most significantly up-regulated in Cx43-KO-S1 cells with fold changes of 5, 4 and 3.6, respectively. Hsa_circ_0001072 (originating from *GTDC1* gene) and hsa_circ_0084765 (originating from *EYA1* gene) were most significantly down-regulated in Cx43-KO-S1 cells with a fold change of 4 (Fig. 2b). Most circRNAs were transcribed from chr1, chr2, chr7, chr11, chr12, chr15, chr16 and chr17, but rarely from chr18 and chrX (Fig. 2c). Hierarchical clustering of the 121 significantly dysregulated circRNAs resulted in separate clustering of pretumorigenic and nontumorigenic cells (Fold Change > 2 and adjusted p-value < 0.05). Of these circRNAs, 75 were up-regulated (62%) and 46 down-regulated (38%) (Fig. 2d). Moreover, 18 significantly dysregulated circRNAs (ten up-regulated and eight down-regulated) were selected for validation (Table 1a) based on whether the genes from which they originate were involved in cancer-related and/or epithelial polarity pathways. Furthermore, since all detected circRNAs were differentially expressed due to the loss of Cx43 in the cultured epithelia, all three circRNA isoforms originating from the Cx43 (*GJA1*), were additionally chosen for validation (Table 1b).

**Table 1:**
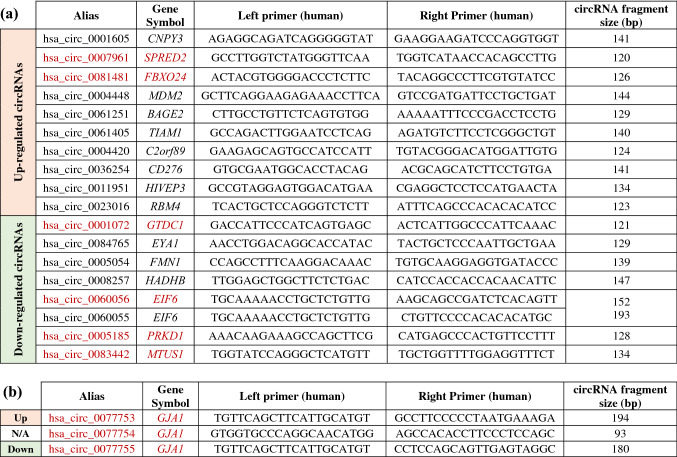
Selection of 18 circRNAs involved in cancer-related and/or epithelial polarity pathways for RT-qPCR validation.

(a) Ten up-regulated (of 75 significantly up-regulated) and eight down-regulated (of 46 significantly down-regulated) circRNAs in Cx43-KO-S1 as compared to S1 cells were chosen for further validation based on whether the genes from which they originate were involved in cancer-related and/or epithelial polarity pathways. (b) The three isoforms of circRNAs that originate from Cx43 (*GJA1*) mRNAs were also chosen due to their relevance to the 3D risk-progression culture model. One Cx43 circRNA isoform, hsa_circ_0077754, was not detected by the microarray due to its short size of 93 bp, since library size-selections usually excludes circRNAs < 200 nt long^58^. Alias represents the circRNAs probe name as annotated by CircBAse^59^. Gene Symbol denotes the gene of the transcript from which this circRNAs originate. Primers were designed using CircularRNA Interactome in silico tool^60^ and purchased from Eurofins (Canada). The circRNA fragment size represents the size of the resulting amplified PCR fragment. The circRNAs highlighted in red font were significantly dysregulated in Cx43-KO-S1 as compared to S1 cells in 3D, as validated through RT-qPCR (shown in Fig. 4).

### RT-qPCR validation of the 18 selected circRNAs revealed two significantly up-regulated and seven significantly down-regulated circRNAs associated with the loss of Cx43

**Figure 3:**
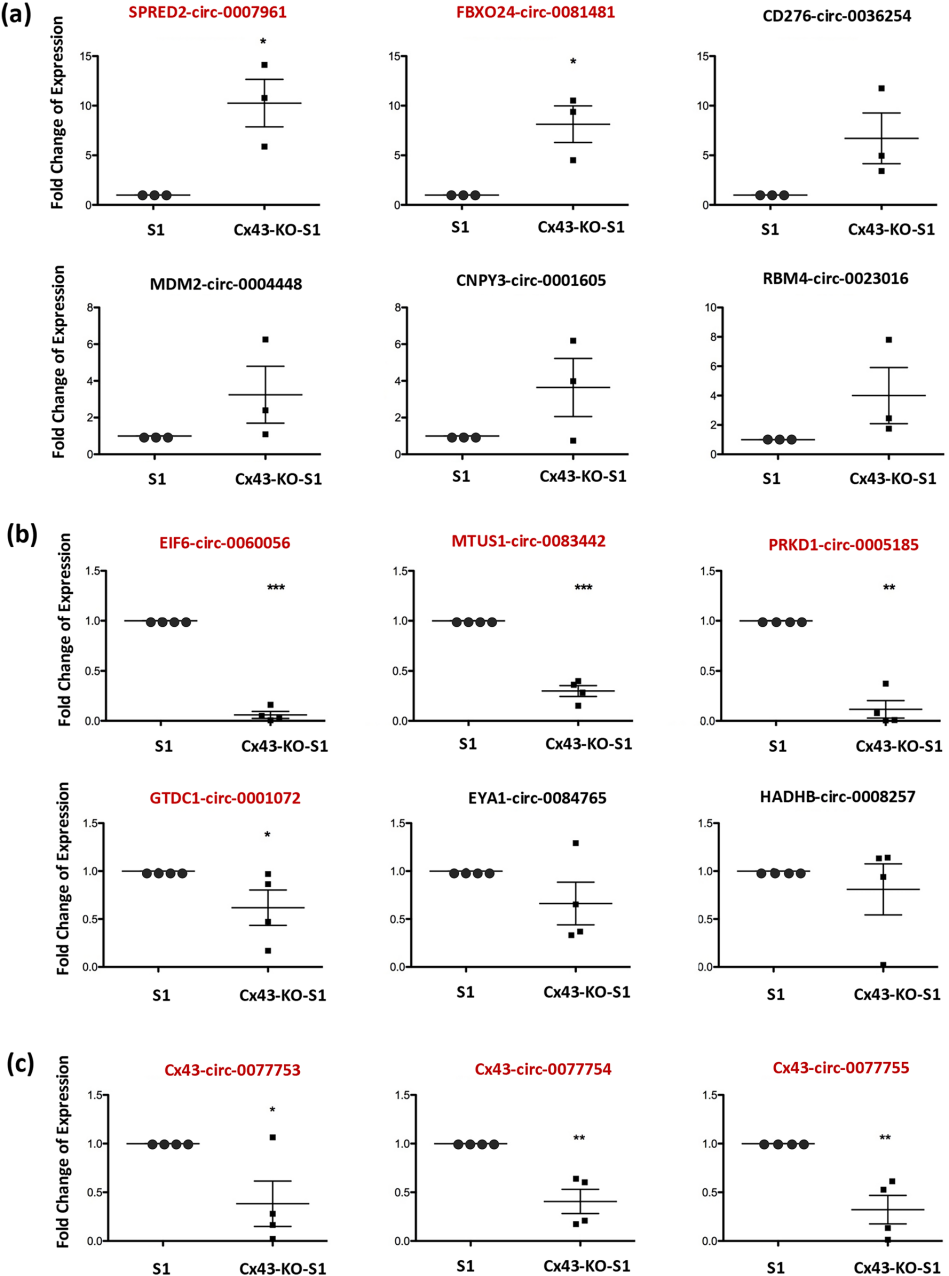
RT-qPCR validated nine significant differentially expressed circRNAs in the cultured epithelia. Four replicates of Cx43-KO-S1 and four replicates of S1 cells were plated in Matrigel for 11 days. Total RNA was extracted and RT-qPCR was performed in Cx43-KO-S1 versus S1 breast epithelial cells in 3D using 18S ribosomal RNA as an endogenous control for (**a**) the selected up-regulated circRNAs and (**b**) the selected down-regulated circRNAs and (**c**) the three Cx43 (*GJA1*) derived circRNAs as per microarray results. Dot plot represents the mean fold change with the standard error of mean as error bars of each circRNA expression in the breast epithelial acini in 3D. The circRNAs highlighted in red font were confirmed to be significantly dysregulated in Cx43-KO-S1 as compared to S1 cells in 3D. *denotes p < 0.05 and **denotes p < 0.01 and *** denotes p < 0.001 for Cx43-KO-S1 versus S1 cells using one-tailed unpaired T-test.

Real time polymerase chain reaction (RT-qPCR) was performed on three to four replicates of acini obtained from Cx43-KO-S1 and S1 breast epithelial cells in 3D. 18S ribosomal RNA was used as an endogenous control. Two of the ten tested up-regulated circRNAs, hsa_circ_0007961 (originating from *SPRED2*) and hsa_circ_0081481 (originating from *FBXO24*) were confirmed to be significantly upregulated in Cx43-KO-S1 compared to S1 cells in 3D culture (Fig. 3a). Four of the eight tested down-regulated circRNAs, hsa_circ_0060056 (from *EIF6*), hsa_circ_0083442 (from *MTUS1*), hsa_circ_0005185 (from *PRKD1*) and hsa_circ_0001072 (from *GTDC1*) were confirmed to be significantly down-regulated in Cx43-KO-S1 versus S1 breast epithelial cells in 3D (Fig. 3b). The rest of the chosen circRNAs still showed the same expected regulation pattern (up or down) as detected in microarrays, however it was not significant, likely due to the slightly high false discover rate (FDR) for these circRNAs from the microarray analysis. Thus, their data was not presented here and were not studied further. Moreover, all three circRNA isoforms originating from Cx43 (*GJA1*) gene, hsa_circ_0077753, hsa_circ_0077754 and hsa_circ_0077755 were confirmed through RT-qPCR to be significantly down-regulated in Cx43-KO-S1 versus S1 cells (Fig. 3c). The next step was to link these circRNAs sponges to their target (predicted and actual) miRNAs.

### miRNA sequencing revealed 65 differentially expressed miRNAs in Cx43-KO-S1 versus S1 acini specific to Cx43 loss

**Figure 4:**
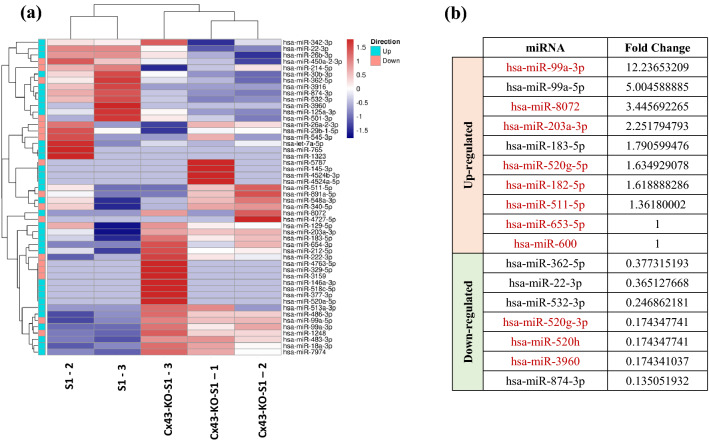
Sequencing revealed 29 significantly up-regulated and 36 significantly down-regulated mature miRNAs in Cx43-KO-S1 cells as compared to S1 cells in response to Cx43 silencing in cultured epithelia. Triplicates of Cx43-KO-S1 and triplicates of S1 cells were plated on Matrigel™ for 11 days. Total RNA was extracted, reverse transcribed and hybridized for sequencing using Illumina’s NovaSeq6000. (**a**) A heat map of unsupervised hierarchical clustering analysis shows for simplicity only miRNAs that were significantly detected from miRNA sequencing data (Fold Change > 2) and are in common with some of the five top MREs for each of the 121 significant differentially expressed circRNAs as predicted by Arraystar's miRNA target prediction software. Red depicts up-regulated miRNAs and blue depicts down-regulated ones in pretumorigenic Cx43-KO-S1 cells compared to nontumorigenic S1 counterparts. Samples were clustered using hierarchical clustering and miRNAs were similarly clustered using hierarchical clustering and are annotated with the direction (up or down-regulation) of the associated circRNAs in Cx43-KO-S1 samples versus S1 samples. Bright blue boxes annotate miRNAs that are predicted to bind to up-regulated circRNAs, whereas pink boxes annotate those associated with circRNAs that are down-regulated in Cx43-KO-S1 as compared to S1 acini. (**b**) A table showing the regulation pattern of miRNAs from miRNA sequencing of the 3D culture model that are in common with predicted MREs of only the 18 chosen circRNAs (from Tables 2, 3), Fold change ≥ 1. The miRNAs in red font represent common significant miRNAs from miRNA sequencing results of 3D culture model and MREs that can be sponged by the nine validated circRNAs through RT-qPCR. Thus, these were selected for investigation in the potential post-transcriptional signature axes in the scope of this paper.

After identifying the dysregulated circRNAs in response to Cx43 silencing in the cultured epithelia, their miRNA expression profile was studied. Focusing on the sponging activity of circRNAs to their target miRNAs, triplicates of the same 3D drip culture samples were submitted for miRNA sequencing. This was performed to compare dysregulation of actual miRNAs in the cultured epithelia detected through sequencing to the miRNA response elements, MREs, predicted to be “sponged” by the significant circRNAs (shown in Table 3) based on Arraystar's miRNA target prediction software^52,53^. Sponging refers to circRNAs exhibiting endogenous competing binding sites (one to several) for each specific target miRNA. They covalently bind the mature target miRNA, downregulating its expression, hence preventing it from binding its own target mRNAs and repressing translation^29^. miRNA sequencing revealed 29 significantly up-regulated (44.6%) and 36 significantly down-regulated (55.4%) mature miRNAs in response to Cx43 silencing in the cultured epithelia. Heat map of hierarchical cluster analysis was used to depict only the differential expression patterns of miRNAs that were both (i) significantly detected in miRNA sequencing and (ii) predicted to be sponged by (one or more of) all the 121 circRNAs significantly dysregulated in microarray of the same model (Fig. 4a). However, very few reads mapped to miRNAs (nearly all miRNAs had zero counts) for sample S1 − 1 Control, and therefore this sample was excluded from any of the analyses going forward. For better selectivity, actual differentially expressed miRNAs from 3D culture model sequencing that are in common with predicted MREs that can be sponged by one or more of only the 18 selcted circRNAs were tabulated in Fig. 4b, and the MREs sponged by the nine validated circRNAs were highlighted. Of these, eight miRNAs, miR-99a-3p, miR-8072, miR-203a-3p, miR-520g-5p, miR-182-5p, miR-511-5p, miR-653-5p and miR-600 were significantly up-regulated while three miRNAs, miR-520g-3p, miR-520h and miR-3960 were significantly down-regulated in Cx43-KO-S1 versus S1 cells, and were presumably sponged by one or more of the nine validated circRNAs by RT-qPCR (shown in Fig. 3). Thus, these miRNAs were further considered to function in the potential post-transcriptional axes that might be dysregulated as a result of Cx43 loss in the breast epithelium, and in turn, potentially play a role in heightened risk of breast cancer initiation.

### Fifteen tumor-associated miRNAs from microarrays of a validation cohort of early-stage breast cancer patients exhibited reported involvement in epithelial polarity and cancer-related pathways

Nassar et al.^47^ previously identified 74 dysregulated miRNAs in microarrays from 45 invasive ductal carcinoma (IDC) versus 17 normal adjacent breast tissues from the Lebanese population (Fold Change > 2) and compared their profile to 197 American breast cancer patients and 87 normal samples from TCGA (The Cancer Genome Atlas), with matched stage, histology and metastasis status^47^. All the Lebanese patient cohort were estrogen receptor (ER) positive, 97.8% were progesterone receptor (PR) positive and 24.5% had human epidermal growth factor receptor 2 (HER2) over-expression. The majority of the tumors were of grade 2, and half of them presented with lymph node involvement. Moreover, 42.2% were below the age of 40 and none of the patients had distant metastasis, thus this cohort was categorized into early-stage breast cancers. We uncovered among these miRNAs through a comprehensive literature review fifteen tumor-associated miRNAs involved in early events of breast tumorigenesis that contribute to loss of acinar morphogenesis^49,61–64^. Among these miRNAs, miR-200c, miR-492, miR-638, miR-663, miR-2861, miR-3960, miR-183, miR-182) were commonly up-regulated in both Lebanese and US patients while miR-492 and miR-663 were exclusive to the Lebanese population. Seven tumor-associated miRNAs, miR-145, miR-125b, miR-100, miR-139-5p and miR-99a, were commonly down-regulated in both Lebanese and US populations (Table 2). Importantly, miR-3960, miR-183, miR-182, miR-145 and miR-99a were in common between patient miRNAs involved in epithelial polarity and MREs predicted to be sponged by the 18 selected circRNAs (as shown in Tables 1 and 3). However, only miR-3960 and miR-182 belonged to MREs that can be sponged by the validated circRNAs confirmed through RT-qPCR. Thus miR-3960 and miR-182 were considered to function in the potential post-transcriptional axes that might be dysregulated as a result of Cx43 loss in the breast epithelium, and in turn, potentially play a role in heightened risk of breast cancer initiation.

**Table 2:**
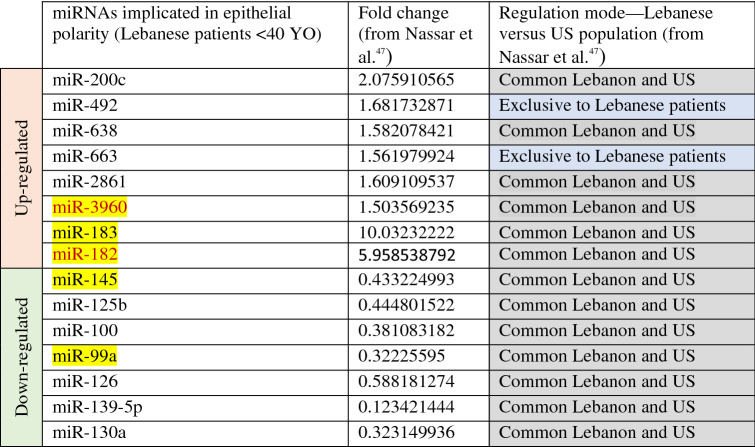
Literature search of tumor-associated miRNAs from microarrays of early-stage Lebanese breast cancer patients^47^ uncovered fifteen miRNAs involved in epithelial polarity and cancer pathways.

Building on Nassar et al.^47^, 74 miRNAs were differentially expressed in Lebanese patients with early-stage breast cancer with no distant metastasis, of which 42.2% were below the age of 40. We identified through a comprehensive literature review 15 patient tumor-associated miRNAs that are involved in early events of breast tumorigenesis and affect epithelial polarity^49,61–64^. Eight of these miRNAs are up-regulated and seven are down-regulated in breast cancer tissues as compared to normal adjacent tissues. Orange highlight panel refers to up-regulated miRNAs, green highlight panel represents down-regulated miRNAs, blue highlights refer to miRNAs exclusively dysregulated in the Lebanese population, while miRNAs highlighted in grey refer to miRNAs commonly dysregulated in both Lebanese and matched US patients (stage, histology and metastasis statuses) as reported in Nassar et al.^47^. miRNAs highlighted in yellow are in common between early-stage patient miRNAs involved in epithelial polarity and MREs predicted to be sponged by the selected significant circRNAs (as shown in Tables 1 and 3). Only miRNAs highlighted in both yellow highlight and red font are implicated in epithelial polarity from patients miRNome and are in common with MREs predicted to be sponged by the RT-qPCR validated significant circRNAs. Thus, these were selected for investigation in the potential post-transcriptional signature axes in the scope of this paper.

**Table 3:**
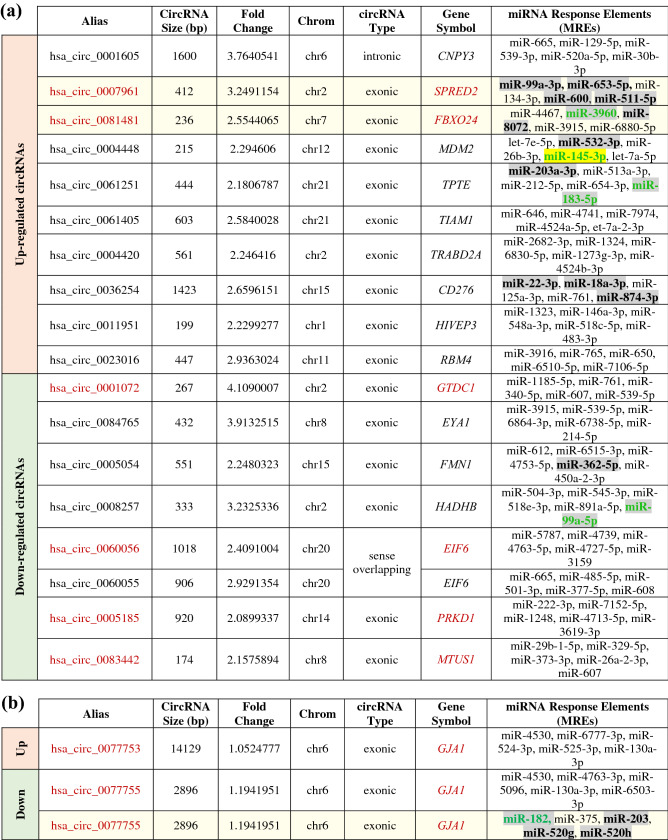
Expression and detection of selected circRNAs (cells microarray) and their target miRNAs (cells sequencing and patients microarray^47^) identified three potential risk-assessment axes for breast cancer initiation.

(a) Listing of the eighteen dysregulated circRNAs chosen for further validation. (b) Listing of two of the three isoforms of circRNAs that originate from Cx43 (*GJA1*) mRNAs that were detected in the microarray (hsa_circ_0077754 was not detected due to its short size). Alias represents circRNAs name as annotated in CircBAse^59^. CircRNAs Size indicates the mature spliced size of circRNAs, Fold Change denotes the absolute ratio (no log scale) of normalized intensities between the two cell lines and Chrom represents the chromosomes that from which each circRNA originates. For circRNAs Type, exonic represents circRNA arising from an exon, intronic arising from an intron and sense overlapping arising from the same gene locus as the linear transcript, but neither exonic nor intronic. Gene Symbol denotes the gene from which circRNA originates. MREs represent the predicted miRNA response elements by Arraystar. The MREs highlighted in grey were shown to be differentially expressed in miRNA sequencing results of Cx43-KO-S1 versus S1 breast epithelial cells in 3D (shown in Fig. 3). The MREs in green font were found dysregulated in microarrays from the early-stage Lebanese breast cancer validation patient cohort by Nassar et al.^47^ and involved in cancer initiation events (shown in Table 1). MREs highlighted in yellow are involved in breast cancer as described in Naser Al Deen et al.^8^. The circRNAs in red font were confirmed to be significantly dysregulated in Cx43-KO-S1 as compared to S1 cells in 3D through RT-qPCR (shown in Fig. 4). The three rows highlighted in light yellow represent the three potential mRNA-circRNA-miRNA breast cancer risk-assessment axes that will be discussed further. These were the only axes with validated circRNAs (RT-qPCR) and with predicted MREs that were also dysregulated in the miRNomes from the cultured epithelia and/or early-stage patients. All MREs were predicted using Arraystar's miRNA target prediction software^52,53^, except for the last row in (b), where circRNAs Interactome^60^ was used in addition to predict MREs as published in Naser Al Deen et al.^8^ (as shown to be involved in breast cancer initiation pathways and circRNA axes originating from Cx43).

### Three mRNA-circRNA-miRNAs axes were proposed, and one axis was validated with potential for risk-assessment of breast cancer initiation

**Figure 5:**
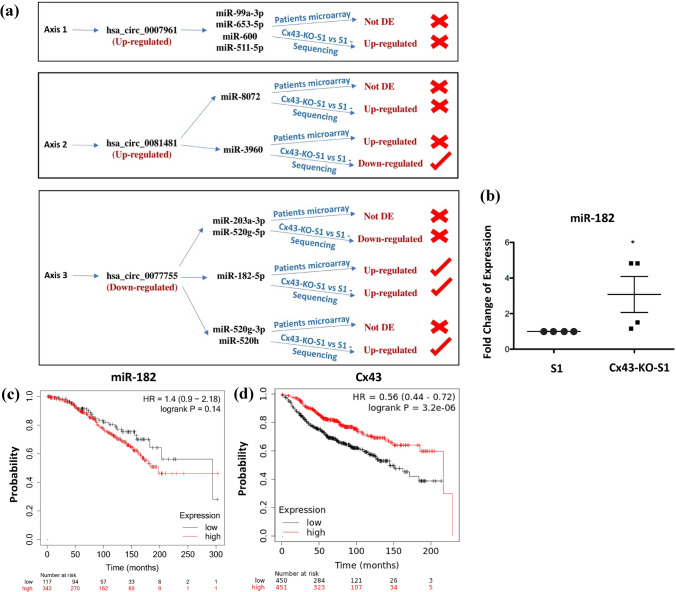
Selection of one validated mRNA-circRNA-miRNA breast cancer initiation risk-assessment axis. (**a**) Comparative flow chart representing the dysregulation patterns of the validated circRNAs and that of their target miRNAs, based on (i) miRNA sequencing in Cx43-KO-S1 cells compared to S1 cells (shown in Fig. 3) and (ii) tumor-associated miRNAs from microarrays of early-stage Lebanese breast cancer patient cohort as reported in Nassar et al.^47^ (shown in Table 1). Only Cx43/has_circ_0077755/miR-182 axis exhibits the expected inverse dysregulation pattern between circRNA and their target miRNAs in both cells and patients (when circRNA is down-regulated, its MRE should be up-regulated, and vice versa). (**b**) RT-qPCR further confirmed the upregulation of miR-182 in four samples of Cx43-KO-S1 cells as compared to S1 counterparts using RNU6B as an endogenous control. * denotes a p.value < 0.05 for Cx43-KO-S1 versus S1 cells using one-tailed unpaired T-test. (**c**) Using METABRIC breast cancer miRNA dataset in the Kaplan–Meier Plotter^50^, the survival analysis for miR-182 in 460 patients with grade II breast tumors was plotted. miR-182 seems to associate with poor prognosis when up-regulated in grade II breast tumors. (**d**) Using all breast cancer mRNA datasets in the Kaplan–Meier Plotter^50,51^, the survival analysis for Cx43 in 901 patients with grade II breast tumors was plotted. Cx43 seems to associate with poor prognosis when down-regulated*.* The same was performed for Grade III breast tumors and presented in (Supplementary Fig. 2a,b), where down-regulation of miR-182 and up-regulation of Cx43 seem to associate with poor prognosis in Grade III breast tumors.

After analyzing, predicting, selecting, validating and comparing significant circRNAs and miRNAs specific to Cx43 silencing in breast epithelial culture and tumor-associated miRNAs from the early breast cancer patient cohort, three potential mRNA-circRNA-miRNA axes were revealed. Focusing on the possible sponging activity of these circRNAs to their target miRNA, the first proposed axis included hsa_circ_0007961 (from *SPRED2)* and its MREs, miR-99a-3p, miR-653-5p, miR-600 and miR-511-5p that were found dysregulated in the breast epithelial culture miRNome. The second proposed axis included hsa_circ_0081481 (from *FBXO24)* and its MREs, miR-3960 (commonly detected in breast epithelial culture and patient miRNome) and miR-8072 (detected in breast epithelial culture miRNome) (Table 3a). The third proposed axis denoted hsa_circ_0077755 (from Cx43 (*GJA1*)) and its MREs, miR-182, which was commonly detected in breast epithelial culture and patient miRNome as well as miR-203, miR-520g and miR-520h, which were only detected in breast epithelial culture miRNome (Table 3b). We next compared the dysregulation pattern of the validated circRNAs in the three potential axes to that of their target MREs that were detected in breast epithelial culture and/or patients miRNomes. Only Cx43/has_circ_0077755/miR-182 axis matched the expected circRNA-miRNA inverse dysregulation levels, suggestive of a possible sponging activity (Fig. 5a). Thus, miR-182 expression level was further confirmed to be significantly up-regulated in four samples of Cx43-KO-S1 cells as compared to S1 counterparts using RNU6B as an endogenous control through RT-qPCR (Fig. 5b). Moreover, survival analysis for miR-182 was generated using METABRIC dataset in in the Kaplan–Meier Plotter^50^. The large patient dataset (460 patients) had long follow-up median of 94 months, was 100% ER positive, 12% HER2 positive, all with grade II tumors with no distant metastasis, and half of them with lymph node involvement, to closely match the characteristics of most of the early-stage Lebanese validation cohort^47^. The results showed that miR-182 seems to associate with poor prognosis when up-regulated in patients with grade II tumors (Fig. 5c). Of note, using all breast cancer mRNA datasets found in the Kaplan–Meier Plotter^50,51^, the survival analysis for Cx43 in 901 patients with grade II breast tumors showed that Cx43 seems to associate with poor prognosis when down-regulated (Fig. 5d). Interestingly, survival analyses of another datasets with the exact characteristics but of grade III breast tumors revealed that miR-182 associates with poor prognosis when down-regulated in grade III tumors of 395 patients (Supplementary Fig. 2a) and that Cx43 associates with poor prognosis when up-regulated in 903 patients with grade III breast tumors (Supplementary Fig. 2b).

### Gene co-expression networks associated with Cx43/hsa_circ_0077755/miR-182 axis correspond to cancer-related pathways

**Figure 6:**
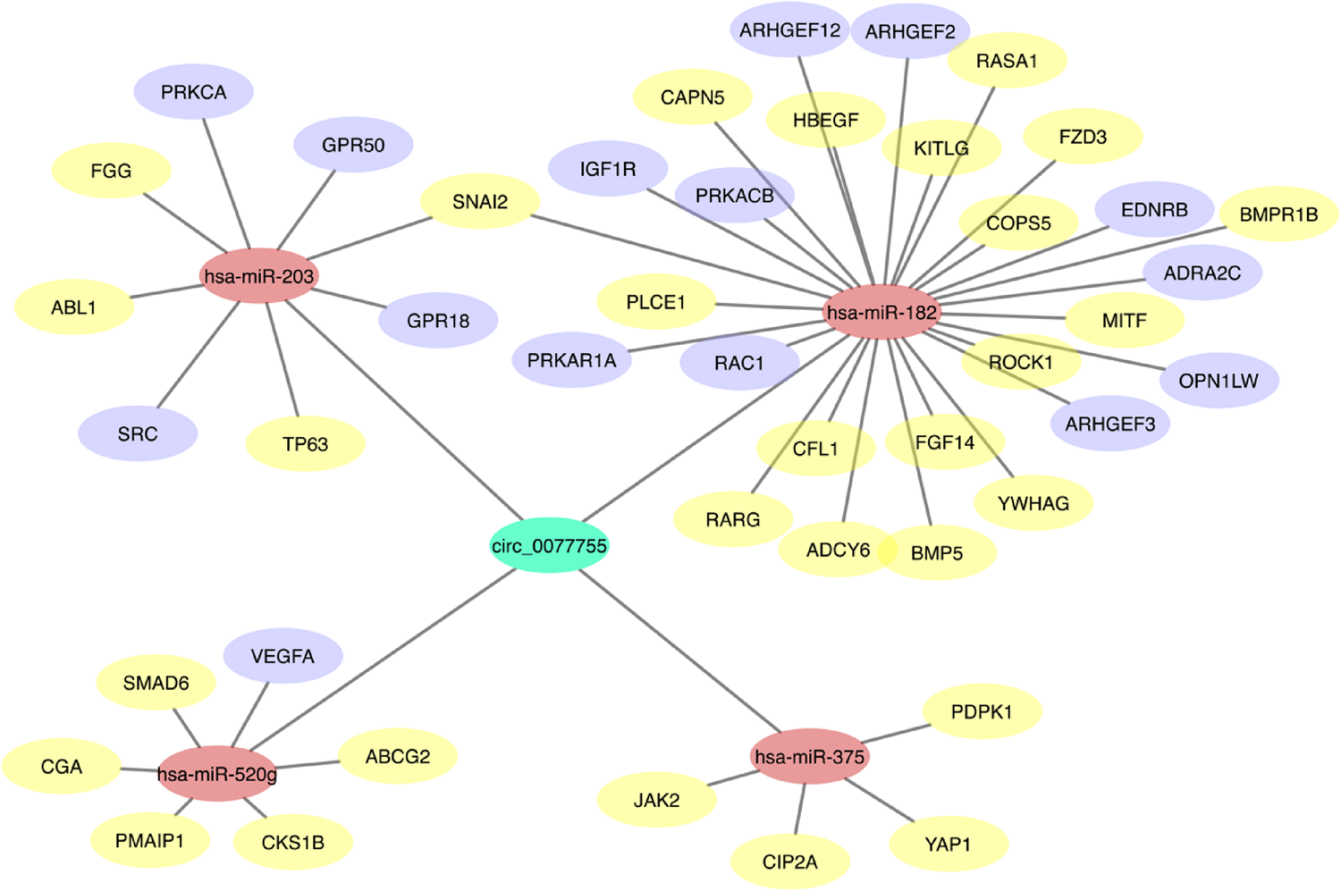
Gene co-expression networks shows the involvement of the validated Cx43/has_circ_0077755/miR-182 axis in cancer-related pathways and in breast cancer. CircRNA-miRNA-mRNA gene co-expression network for hsa_circ_0077755 was predicted by TargetScan^65^ within IPA and Cytoscape was used to draw circRNA-miRNA-mRNA interaction networks. CircRNA is colored in green, miRNAs in pink and mRNAs reported in cancer in yellow and in breast cancer in purple. miR-182 exhibited the largest interaction network with mRNAs involved in cancer-related pathways in the axis.

We opted to further show that the validated Cx43/hsa_circ_0077755/miR-182 axis, spurred from the comparison between patients and the cell culture risk-progression model, is involved in cancer-related pathways. Thus, sequence pairing using Cytoscape software was utilized to link the top predicted MREs (by ArrayStar), which could be sponged by hsa_circ_0077755 to their predicted mRNA targets by TargetScan^65^ within Ingenuity Pathway Analysis (IPA). Only experimentally validated mRNAs involved in cancer-related pathways were kept. The results confirmed that in for hsa_circ_0077755 gene co-expression network, miR-182 exhibited the largest interaction network in cancer-related pathways and in breast cancer pathways and exhibited slight interaction network overlap with miR-203 (Fig. 6).

### Functional analysis for hsa_circ_0077755 is enriched for cancer-related pathways

**Figure 7:**
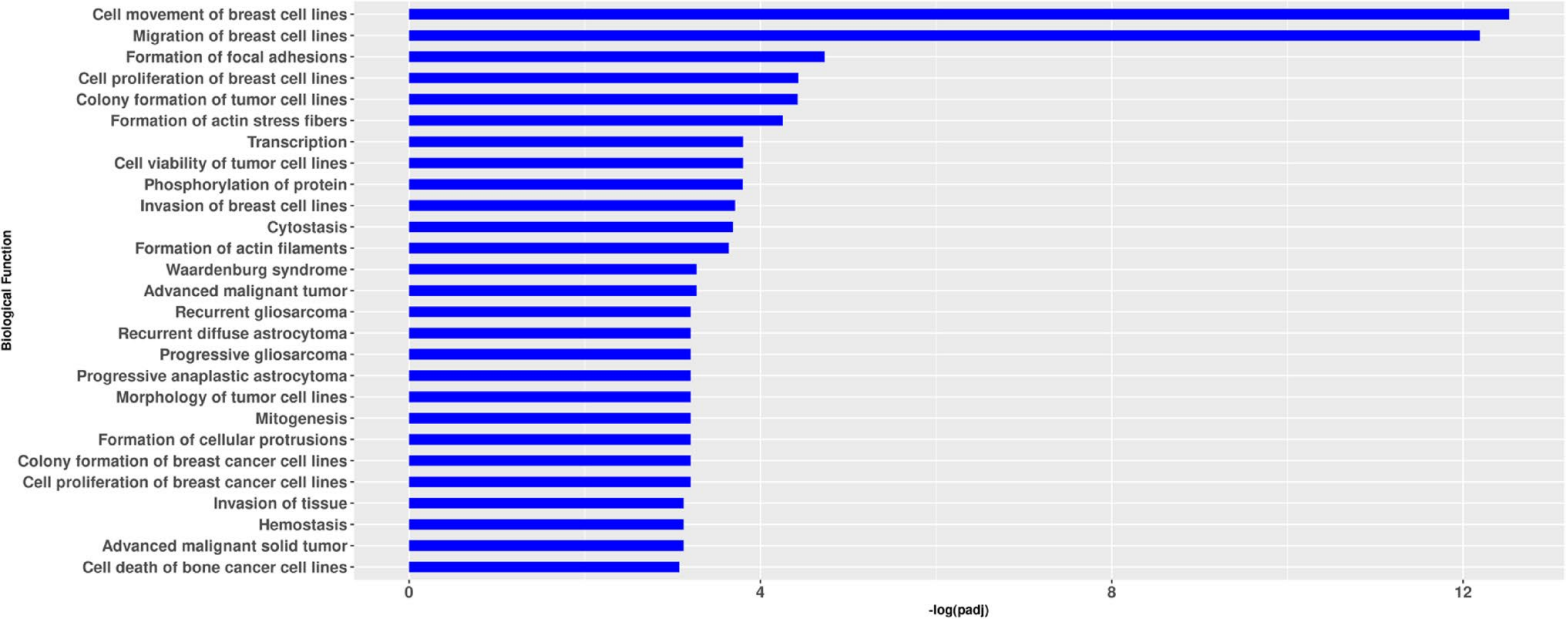
The chosen and validated hsa_circ_0077755 is functionally enriched in cancer-related pathways. A functional enrichment analysis was performed in Ingenuity Pathway Analysis (IPA) of predicted target mRNAs (by TargetScan^65^) for the top five MREs associated with hsa_circ_0077755. P-values were adjusted for multiple testing using the Benjamini–Hochberg method. The ba r plot displays the biological functions on the y-axis and the –log (adjusted p-value) on the x-axis.

Additional in silico tools were used to confirm the involvement of the selected axis in cancer-related pathways. Using IPA (Qiagen), a functional enrichment analysis of the predicted target mRNAs (by TargetScan^65^) for the top five MREs associated with hsa_circ_0077755 was performed (Fig. 7). Hsa_circ_0077755 displayed enrichment of biological functions in cancer with pathways mostly enriched in cell movement, migration and proliferation of breast cell lines, formation of focal adhesions, colony formation and cell viability of tumor cell lines and invasion of breast cancer cell lines. This analysis corroborates the strong involvment of the selected axis in cancer-related pathways.

## Discussion

Recent studies reported miRNAs and circRNAs as active players in tissue homeostasis, tumorigenesis, therapy resistance and metastasis and as novel noninvasive cancer biomarkers^7,8,17,18^. This study brings a novel comprehensive analysis of circRNAs and miRNAs expression axes specific to Cx43 loss and focused on risk assessment of early breast cancer initiation.

### Gap junctions are involved in post-transcriptional regulatory pathways in breast cancer initiation

A main outcome from this study is the demonstration of Cx43 involvement in post-transcriptional regulatory pathways of heightened risk of breast cancer initiation. Upon silencing Cx43 in S1 cells, a total of 121 circRNAs and 65 miRNAs were significantly dysregulated in the pretumorigenic phenotype, adding an important post-transcriptional regulatory layer that Cx43 might play in breast epithelia (Figs. 2 and 4). Our previous findings implicate Cx43 in fundamental aspects of epithelial homeostasis^19^. Specifically, its loss disrupts apical polarity and mitotic spindle orientation, which contributes to cell multilayering, and primes cells for enhanced motility and invasion, depending on the microenvironment^19,20^. These phenotypes represent premalignant mammary lesions (as hallmarks of cancer initiation) like those observed in ductal hyperplasia in a murine model^23^. Recently, we predicted in silico the involvement of Cx43-derived post-transcriptional players as possible biomarker signatures for the risks of early breast cancer. Three Cx43-derived circRNA isoforms (hsa_circ_0077753, hsa_circ_0077754, and hsa_circ_0077755) and a panel of their predicted sponged miRNAs (miR-182 miR-375, miR-203, miR-520g and miR-520h)^8^ were proposed to recapitulate phenotypes caused by Cx43 loss when dysregulated^14,37,48,49^. For instance miR-182, miR-203 and miR-375 were up-regulated during breast lobular neoplasia progression and correlated with loss of breast cellular organization and development of hyperplastic phenotypes^49^.

In this study, we specifically aimed to validate the actual post-transcriptional axes that might be involved in regulatory pathways contributing to heightened risk of breast cancer initiation upon Cx43 loss. Notably, the choice of validated circRNAs and miRNAs in the axes was dependent on their involvement in pathways that recapitulate phenotypes observed upon loss of Cx43 (Table 1). miR-99a was up-regulated in Cx43-KO-S1 compared to S1 cells. Turcatel et al.^66^ reported an involvement of miR-99a in epithelial to mesenchymal transition (EMT) when up-regulated in murine mammary gland, and in driving the progression of breast cancer through cell migration and invasion by regulating TGF-β and affecting the phosphorylation of SMAD3. miR-3960, which was commonly up-regulated in early breast cancer Lebanese and US patients (matched for stage, histology and metastasis statuses)^47^, and down-regulated in Cx43-KO-S1 compared to S1 cells was linked to breast cancer-mediated bone metastasis via Runx2/miR-3960/miR-2861 axis^67^. Moreover, miR-182, which was commonly up-regulated in early-stage Lebanese and US patients and in Cx43-KO-S1 compared to S1 cells, was up-regulated in various human breast cancer subtypes and acted as an oncogene^68^. Over-expression of miR-182 in vitro enhanced cell migration, invasion and proliferation and increased tumor volume and lung metastasis in vivo by regulating FOXF2^69^. In addition, in an attempt to identify insults that lead to breast cancer initiation, Duforestel et al. revealed that exposing non-neoplastic MCF10A cells to low pressure but sustained DNA hypomethylation, via the TET pathway, through repeated exposure to Glyphosate, in combination with over-expression of miR-182 primed tumor-initiation from these non-neoplastic cells in vivo^70^. Thus, desponging these miRNAs by the differentially expressed circRNAs specific to Cx43 loss might drive mammary pretumorigenic phenotypes and cancer initiation, and hence, corroborate gap junction’s involvement in post-transcriptional regulatory axes that heighten the risk of breast cancer initiation.

### One mRNA-circRNA-miRNAs axis acts as potential biomarker signatures for heightened-risk of breast cancer initiation

Only few circRNA biomarker axes have been reported in breast cancers^39–45^, but none specific to pre-malignant epithelial polarity transitions that might increase the risk of initiation of the disease. For instance, hsa_circ_0001982 was significantly up-regulated in vivo and in vitro, and its knock-down inhibited proliferation and invasion and promoted apoptosis in breast cancer cells by targeting miR-143^39^. Hsa_circ_0008039/miR-432-5p/E2F3 exhibited oncogenic roles in breast cancer and suppressing hsa_circ_0008039 inhibited proliferation and migration and arrested cell cycle through targeting miR-432-5p, in turn targeting E2F3^41^. Circ-Dnmt1 was up-regulated in breast cancer patient samples and in eight cell lines and could bind to oncogenic proteins p53 and AUF1, exhibiting oncogenic potential^43^. Hsa_circ_0072309 over-expression dramatically inhibited proliferation, migration and invasion of breast cancer cells in vitro and repressed breast cancer growth in vivo through sponging miR-492, serving as a prognostic biomarker^44^ while hsa_circ_0001785 served as a potential plasma diagnostic marker^45^.

We investigated potential post-transcriptional axes specific to Cx43 loss that might heighten the risk for breast cancer initiation. The first validated up-regulated hsa_circ_0007961 (from *SPRED2*) was predicted to sponge miR-653-5p, miR-99a-3p, miR-134-3p, miR-600 and miR-511-5p (Table 3a). Of note, miR-653-5p, miR-99a-3p, miR-600 and miR-511-5p were found dysregulated in miRNA sequencing of the cultured epithelia specific to Cx43 loss (Fig. 4b) However, none of these miRNAs matched the expected circRNA-miRNA inverse dysregulation levels suggestive of a possible sponging activity, and neither of the miRNAs were detected in the early-stage patient validation cohort miRNome. This axis was hence not validated (Fig. 5a). The second up-regulated circRNA in our selection, hsa_circ_0081481 (from *FBXO24*), was predicted to sponge miR-3960, miR-4467, miR-8072, miR-3915, miR-6880 (Table 3a). Of these miRNAs, miR-8072 was found upregulated in miRNome of the cultured epithelia while miR-3960 was found down-regulated in the cultured epithelia and matched the expected circRNA-miRNA inverse dysregulation levels. However, miR-3960 was found up-regulated in the patient validation cohort, contrary to its expected levels, and thus the second axis was also not validated (Fig. 5a). As for the down-regulated circRNA, hsa_circ_0077755 (originating from Cx43 (*GJA1*), the gene of interest of our previous and current studies^8,13,14^), all of the MREs it could sponge, miR-182, miR-203, miR-520g, and miR-520h (except for miR-375) were detected in the miRNome of the cultured epithelia (Table 3b). However, only miR-182 was (i) found up-regulated in the cultured epithelia, (ii) found similarly up-regulated in the patient validation cohort and (iii) matched the expected circRNA-miRNA inverse dysregulation levels (Fig. 5a). Thus Cx43/hsa_circ_0077755/miR-182 axis was the only validated biomarker axis for heightened-risk of breast cancer initiation.

### Cx43/hsa_circ_0077755/miR-182 axis associates with poor prognosis in a differential manner along breast cancer initiation and progression

To further validate Cx43/hsa_circ_0077755/miR-182 axis, RT-qPCR confirmed the significant up-regulation of miR-182 in Cx43-KO-S1 cells compared to S1 counterparts (Fig. 5a). This might imply that the down-regulation of hsa_circ_0077755, which is indicative of loss of of Cx43 mRNA expression along breast cancer initiation^8^, relieves its sponging activity on miR-182 causing an upregulation in its expression, which in turn acts as an oncogene and primers tumor initiation as mentioned earlier^68–70^. In addition, survival analyses for patients with grade II breast tumors revealed poor prognostic association upon the up-regulation of miR-182 in a cohort of 460 patients (Fig. 5c) and down-regulation of Cx43 in a cohort of 901 patients (Fig. 5d). Interestingly, when performed on patients with grade III breast tumors with the exact characteristics, survival analyses revealed poor prognostic association upon the down-regulation of miR-182 in a cohort of 395 patients (Supplementary Fig. 2a) and the up-regulation of Cx43 in a cohort of 903 patients (Supplementary Fig. 2a)^50,51^. Indeed, low levels of Cx43 in the primary breast tumors at initial stages associate with poor prognosis^18^, while high levels of Cx43 in breast cancer patients biopsied at later tumor stages is associated with poor prognosis and is suggestive of enhanced tumor progression and invasion^71^. This is since the tumor epithelial cells up-regulate the expression of gap junction intercellular communication with endothelial cells, facilitating intravasation and extravasation in order to invade and metastasize^72^. Thus, Cx43 acts as a tumor suppressor, its loss during early tumorigenesis and early stages of the malignancy promotes breast cancer initiation^19^, progression^20^ and associates with poor prognosis. At later tumor stages, Cx43 re-expression facilitates invasion and metastasis and associates with poor prognosis^72^. Thus, Cx43 (tumor suppressor) down-regulation and miR-182 (oncogene) up-regulation at early tumorigenic stage seem to associate with poor prognosis, while at later advanced tumoral stages, Cx43 up-regulation and hence miR-182 down-regulation associate with poor prognosis, as shown by the validation datasets.

## Conclusion

Cx43/hsa_circ_0077755/miR-182 is the only validated post-transcriptional axis specific to Cx43 loss that might serve as a biomarker predictor of heightened risk of breast cancer initiation (Fig. 1). Moreover, Cx43/hsa_circ_0077755/miR-182 axis differential dysregulation pattern associates with prognosis along breast cancer initiation and progression. We previously proposed a possible biomarker signature of Cx43 mRNA-circRNAs-miRNAs axes for detection and prevention of early-onset breast cancer^8^, which parallel roles that Cx43 plays along breast tumorigenesis. Here, we confirm the involvement of Cx43 in post-transcriptional regulatory axes in breast cancer initiation, match the miRNA dysregulation pattern to an early-stage breast cancer patient cohort and propose Cx43/hsa_circ_0077755/miR-182 as a biomarker axis of heightened risk of breast cancer initiation.

### Limitations and future directions

CircRNAs can sponge tens of different miRNAs, and each miRNA can target hundreds of mRNAs, and hence might affect multiple downstream functional pathways^59,60,73^. Thus, it is expected that other pathways might be implicated in the chosen validated axis. However, the 3D culture model that recapitulates heightened risks for cancer development helped us narrow-down these pathways to those downstream (or parallel) to Cx43 loss, since the two cell lines are identical, except for silencing Cx43. Thus (i) by using this breast cancer risk progression culture model and (ii) by only choosing miRNAs that matched miRNAs from microarrays of early-stage Lebanese breast cancer population (that are at most heightened risk of developing this early malignancy^2^), circRNAs and miRNAs selection was carefully chosen to represent what might be reflected in heightened-risk of breast tumors initiation. Future studies should validate the proposed direct interactions between Cx43 and hsa_circ_0077755 and miR-182 by overexpressing hsa_circ_0077755 and examining its effect on miR-182 and Cx43 in this 3D culture model. Notably, studies by our group are underway to investigate whether candidate miRNA over-expression, including miRNA-182/183 cluster and other Lebanese-specific dysregulated miRNAs, might recapitulate tumor-initiation phenotypes seen upon Cx43 loss. Finally, studies should validate this axis in sera of breast cancer-free controls and those at risk and in early-stage and advanced-stage breast cancer patients to test their potential role as noninvasive biomarkers of risk-assessment of initiation and prognosis, respectively.

## Methods

### Three-dimensional cell culture

Nontumorigenic HMT-3522 S1 (S1) human breast epithelial cells^46^ between passages 52 and 60, were routinely maintained as a monolayer on plastic (2D culture) in chemically defined serum-free H14 medium^56,74^ at 37 °C and 5% CO_2_ in a humidified incubator. H14 medium was changed every 2–3 days. For 2D cultures, cells were plated on plastic substrata at a density of 2.3 × 10^4^ cells/cm^2^. The drip method of 3D culture was used to induce the formation of acini. Briefly, cells were plated on Matrigel (50 μl/cm^2^; BD Biosciences, 354234) at a density of 4.2 × 10^4^ cells/cm^2^ in the presence of culture medium containing 5% Matrigel^56,75^. The EGF was omitted from the culture medium after day 7 to allow completion of acinar differentiation (usually observed on day 8 or 9)^56^. Cx43 was down-regulated in S1 cells via retroviral delivery of shRNA, as described by Bazzoun et al.^19^.

### Total RNA isolation and quality control (QC)

Total RNA from cells in 3D culture was extracted using TRIzol reagent (Invitrogen, Carlsbad, CA, USA) following the manufacturer’s protocol. Purity and concentration of RNA samples were examined spectrophotometrically by absorbance measurements at 260, 280 and 230 nm using the NanoDrop ND-1000 (Thermo Fisher Scientific, Wilmington, DE, USA). OD260/OD280 ratios between 1.8 and 2.1 were deemed acceptable. Note, same biological 3D culture samples were used for both the circRNA microarrays and the miRNA sequencing to insure consistency.

### Sample preparation and hybridization for circRNAs microarrays

Sample labeling, and array hybridization were performed according to the manufacturer’s protocol (Arraystar Inc.), as previously described by Zhang et al.^76^. Briefly, total RNAs were digested with RNase R (Epicentre, Inc.) to remove linear RNAs and enrich circRNAs. Then, the enriched circRNAs were amplified and transcribed into fluorescent cRNA utilizing a random priming method (Arraystar Super RNA Labeling Kit; Arraystar). Labeled cRNAs were purified by RNeasy Mini Kit (Qiagen). Concentration and specific activity of labeled cRNAs (pmol Cy3/μg cRNA) were measured by NanoDrop ND-1000. One μg of each labeled cRNA was fragmented by adding 5 μl 10× Blocking Agent and 1 μl of 25× Fragmentation Buffer, then the mixture was heated at 60 °C for 30 min, finally 25 μl 2× Hybridization Buffer was added to dilute the labeled cRNA. 50 μl of Hybridization Buffer was dispensed into the gasket slide and assembled to the circRNA expression microarray slide. Slides were incubated for 17 h at 65 °C in an Agilent Hybridization Oven.

### Data processing and analysis for circRNAs microarrays

Agilent Feature Extraction software (version 11.0.1.1) was used to analyze acquired array images (https://www.agilent.com/en/product/mirna-microarray-platform/mirna-microarray-software/feature-extraction-software-228496). Quantile normalization and subsequent data processing were performed using the R software (R Core Team 2019, https://www.R-project.org/)^77^ Limma package. Differentially expressed circRNAs with statistical significance between Cx43-KO-S1 and S1 cells were identified through Volcano Plot filtering and Fold Change (i.e. the ratio of the group averages) filtering. The statistical significance was estimated by t-test. circRNAs having fold changes ≥ 2 and p-values ≤ 0.05 were selected as the significantly differentially expressed. Hierarchical Clustering was performed to show the distinguishable circRNAs expression pattern among samples.

### Annotation for circRNA/miRNA interaction

The circRNA/miRNA interaction was predicted with Arraystar Inc’s home-made miRNA target prediction software based on TargetScan^65^ and miRanda^52^ functions, and the differentially expressed circRNAs within all the comparisons were annotated in detail with the circRNA/miRNA interaction information. Note: CircRNA sequences were predicted by bioinformatics methods following the approach by Salzman et al.^58^.

### miRNA library preparation and sequencing

Triplicate samples of S1 cells and triplicates of Cx43-KO-S1 cells were submitted for small RNA-seq. The Purdue Genomics Facility prepared libraries using the NEXTflex Illumina Small RNA Sequencing Kit v3 (Bioo, Austin, TX) with barcoding performed using UDI primers. A total of 15 PCR cycles were performed. 2 × 50 bp reads were sequenced using the NovaSeq6000. Only one of each read pair was used for analyses, given the short size of miRNAs. Library preparation protocols were modified for the use of unique dual indexes, in order to circumvent index hopping on the Illumina NovaSeq 6000. Before library preparation the RNA quality was checked using an Agilent Nano RNA Chip.

### Heatmap of miRNAs from the breast epithelia that are in common with the MREs sponged by the significant circRNAs

Counts for miRNAs were obtained using miRNA-seq on a NovaSeq 6000. Data were normalized using DESeq2^78^ and then, the log2 of 1 + the normalized counts was scaled by row. Rows were clustered using hierarchical clustering and were annotated with the direction (up- or down-regulation) of the associated circRNAs in Cx43-KO-S1 samples versus S1 control sample using R software (version 3.5.1 https://cran.r-project.org/)^79^. Cutoff points were set for Fold Change > 2 and p.adjusted value ≤ 0.05.

### Functional enrichment of mRNAs associated with circRNA binding miRNAs

A functional enrichment analysis was performed in Ingenuity Pathway Analysis (IPA, QIAGEN Redwood City, http://www.qiagen.com/ingenuity) of predicted target mRNAs (predicted by TargetScan^65^) for the top five predicted sponged miRNAs associated with the chosen circRNAs. Due to the high number of predicted mRNA targets for each miRNA, only mRNAs with strong predicted (based on the cumulative weighted context score of − 0.4 or lower^65^) or experimental evidence were kept in the analysis. Limiting the number of mRNA targets in this way allows for quantification of the significance of the enrichment via a hypergeometric distribution. An enrichment test was performed to determine which biological functions or diseases associated with mRNA targets are observed in association with these mRNAs more often than we would expect by chance.

### RT-qPCR validation of chosen circRNAs (and selection of candidate circRNAs) and divergent primer design

To validate the expression profiles of circRNAs in four replicates of 3D acini of Cx43-KO-S1 versus S1 cells, QuantiTect Reverse Transcription Kit (Qiagen cat # 205311) was used for cDNA Synthesis for 1 μg of RNA per sample according to manufacturer’s protocol (but optimized for the last steps as follows: after the addition of the RNA samples to the reverse-transcription master mix, the reaction (25 μl per reaction) was incubated at 25 °C, 10 min, then at 50 °C, 30 min, then at 85 °C, 5 min). Specific primers (left and right) for each circRNAs were designed using Circular RNA Interactome built-in divergent primer design tool (based on Primer3 Output)^60^ and purchased from Eurofins Genomics (Canada) and were used for the first strand synthesis. 18S ribosomal RNA (TIB Molbiol product no. 1945400 and 1945401) was used as an endogenous control. RT-qPCR was performed as follows: cDNA product was diluted with 75 μl RNase free water. PCR was conducted in a 20 μl reaction volume consisting of the following: 4.0 μl diluted cDNA, 4 μl (total left and right circRNAs primer), 2 μl RNase free water, and 10 μl SYBR Green JumpStart Taq ReadyMix (SIGMA S4438). The RT-qPCR reaction was performed using BioRad CFX96 Real Time System, C1000 Thermal Cycler (Germany) as follows: initial denaturation at 95 °C for 5 min, 40 cycles of amplification at 95 °C for 15 s, annealing and extension at 60 °C for 1 min. Using the ΔΔCq equation, relative expression of the experimental circRNA was determined in the Cx43-KO-S1 samples compared to S1 samples using 18S ribosomal RNA as an endogenous control. Normalization of Cx43-KO-S1 samples was based on S1 samples. Statistical analysis was performed using Prism GraphPad software. One-tailed unpaired t-test was used to compare the circRNA expression in the Cx43-KO-S1 samples versus S1 samples. A p-value < 0.05 was considered statistically significant.

### miRNA expression by quantitative real time-polymerase chain reaction

Reverse transcription of 10 nanograms of the total RNA was performed using the TaqMan MicroRNA Reverse Transcription Kit (Applied Biosystems, USA) according to the manufacturer’s instructions and as previously described by Nassar et al.^47^. Briefly, small nuclear RNA RNU6B, miR-182-5p primers and probes were purchased as part of the TaqMan microRNA Assays Kit (Applied Biosystems, USA) with validated efficiency. cDNA synthesis was carried out for miR-182-5p in each reaction with the endogenous control, RNU6B. RT-qPCR was performed using BioRad CFX96 Real Time System, C1000 Thermal Cycler (Germany). Reactions using 10 μl of 2X TaqMan Universal Master Mix with no Amperase Uracil N-glycosylase (UNG) (Applied Biosystems, USA), 1 μl of the corresponding 20× microRNA probe, 4 μl of DEPC treated water, and 5 μl of cDNA were performed in duplicates for each miRNA probe. cDNA Synthesis and RT-qPCR were repeated twice for each sample and each plate included no reverse transcription control (NRT), no template control (NTC) and normal breast tissue samples. The normalization of the Cx43-KO-S1 was based on the S1 control cells present in the RT-qPCR plate to ensure inter-run calibration. The cycling conditions were 95 °C for 10 min and 40 cycles of 95 °C for 15 s and an annealing temperature of 60 °C for 60 s. Using the ΔΔCt equation, the relative expression of miRNA was determined in the Cx43-KO-S1 samples compared to S1 cells using RNU6B as an endogenous control.

### Cytoscape analysis

CircRNA-miRNA-mRNA gene co-expression network was predicted based on sequence-pairing using Cytoscape software (https://cytoscape.org), version 3.1.0^80^. The top four predicted miRNAs (MREs) associated with circRNAs were used to predict mRNA targets by TargetScan^65^ within IPA. Only mRNAs involved in cancer-related pathways and shown to be targets experimentally or else high confidence predictions by TargetScan were kept. Cytoscape was used to draw circRNA-miRNA-mRNA interaction networks.

## Supplementary Information

Supplementary Information.

## Data Availability

The raw and processed datasets generated from microarray and sequencing analysed during the current study are available on the Geobrowser (to be assigned a number when published).
