# Supplementary material for: A risk progression breast epithelial 3D culture model reveals Cx43/hsa_circ_0077755/miR-182 as a biomarker axis for heightened risk of breast cancer initiation
Source: Sci Rep. 2021 Jan 29;11:2626. doi: 10.1038/s41598-021-82057-y (PMC7846862; doi:10.1038/s41598-021-82057-y)
Supplement: Supplementary file 1 — Supplementary Information. [file 41598_2021_82057_MOESM1_ESM.docx]

**A risk progression breast epithelial 3D culture model reveals Cx43/hsa_circ_0077755/miR-182 as a biomarker axis for heightened risk of breast cancer initiation**

***Nataly Naser Al Deen^1^, Nadia Atallah Lanman^2,3^, Shirisha Chittiboyina^4^, Sophie Lelièvre^2,4^, Rihab Nasr^5^, Farah Nassar^6^, Heinrich zu Dohna^1^, Mounir AbouHaidar^7^*, Rabih Talhouk^1^****

*^1^Department of Biology, Faculty of Arts and Sciences, American University of Beirut,^2^Purdue University Center for Cancer Research, ^3^Department of Comparative Pathobiology and ^4^Department of Basic Medical Sciences, College of Veterinary Medicine, Purdue University, ^5^Department of Anatomy, Cell Biology and Physiological Sciences and ^6^Department of Internal Medicine, Faculty of Medicine, American University of Beirut and ^7^Department of Cell and Systems Biology, University of Toronto. *Corresponding Authors.*

**Abstract**

mRNA-circRNA-miRNAs axes have been characterized in breast cancer, but not as risk-assessment axes for tumor initiation in early-onset breast cancer that is increasing drastically worldwide. To address this gap, we performed circular RNA (circRNA) microarrays and microRNA (miRNA) sequencing on acini of HMT-3522 S1 (S1) breast epithelial risk-progression culture model in 3D and chose an early-stage population miRNome for a validation cohort. Nontumorigenic S1 cells form fully polarized epithelium while pretumorigenic counterparts silenced for gap junction Cx43 (Cx43-KO-S1) lose epithelial polarity, multilayer and mimic premalignant *in vivo* mammary epithelial morphology. Here, 121 circRNAs and 65 miRNAs were significantly dysregulated in response to Cx43 silencing in cultured epithelia and 15 miRNAs from the patient cohort were involved in epithelial polarity disruption. Focusing on the possible sponging activity of the validated circRNAs to their target miRNAs, we found all miRNAs to be highly enriched in cancer-related pathways and cross-compared their dysregulation to actual miRNA datasets from the cultured epithelia and the patient validation cohort. We present the involvement of gap junction in post-transcriptional axes and reveal **Cx43/hsa_circ_0077755/miR-182** as a potential biomarker signature axis for heightened-risk of breast cancer initiation, and that its dysregulation patterns might predict prognosis along breast cancer initiation and progression.

****Address for Correspondence:***

Rabih Talhouk, Ph D, Professor

Department of Biology, Faculty of Arts and Sciences

American University of Beirut, Beirut, Lebanon, P.O. Box: 11-0236

Phone: 00961-1-374374 ext. 3895

Email: [rtalhouk@aub.edu.lb](mailto:rtalhouk@aub.edu.lb)

Mounir G. AbouHaidar, M.Sc., Ph D., (DrE), Professor

Department of Cell & Systems Biology, University of Toronto

25 Willcocks St. Toronto, On. Canada, M5S 3B2

Phone: 416 978 5615, Fax: 416 978 58 78

Email: [mounir.abouhaidar@utoronto.ca](mailto:mounir.abouahidar@utoronto.ca)

**Supplementary Figures**

**(b)**


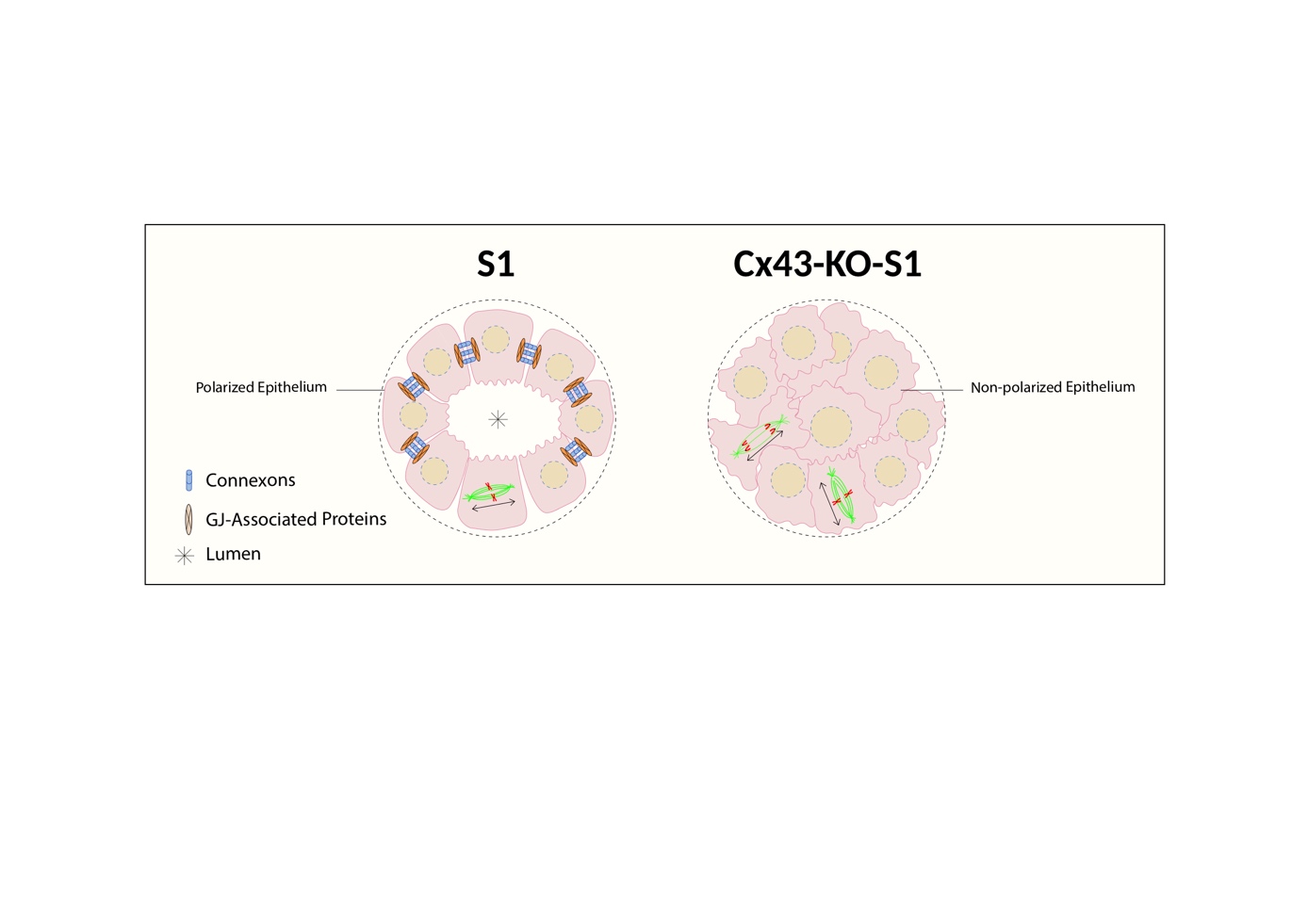


**(a)**

**Supplementary Figure 1. HMT-3522 S1 nontumorigenic and Cx43-KO-S1 pretumorigenic cells in 3D culture as a breast cancer risk-progression model. (a)** Upper panel of immunofluorescence images shows the localization of β-catenin (green) on day 11 in 3D cultures of S1 cells (left panel showing typical apicolateral β-catenin distribution) and in Cx43-KO-S1 cells (right panel showing β-catenin mis-localization upon knockdown of Cx43). Lower panel of immunofluorescence images shows the localization of Cx43 (red) on day 11 in 3D cultures of S1 cells (left panel showing typical apical Cx43 distribution) and in Cx43-KO-S1 cells (right panel showing loss of Cx43 upon knockdown of Cx43). Nuclei were counterstained with DAPI (blue). **(b)** A diagram depicting S1 cells that form phenotypically normal differentiated mammary epithelium (left panel), where the cells assemble around a lumen (
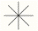
), polarize with apical and basolateral domains and assemble membranous connexons (*
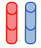
*) with GJ-associated proteins (
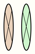
) in GJs between epithelial cells. The down-regulation of Cx43 mRNA levels by knocking-down Cx43 leads to loss of GJ intercellular communication (GJIC), causing loss of communication between neighboring cells, activation of cellular proliferation, disruption of mitotic spindle orientation (MSO), multi-layering, loss of polarity and alteration in polarity protein distribution [19, 20]. MSO (indicated by the double-headed arrows) is depicted based on the directionality of the α-tubulin poles. Proper MSO is shown tangential to the circumference of the growing acinus (
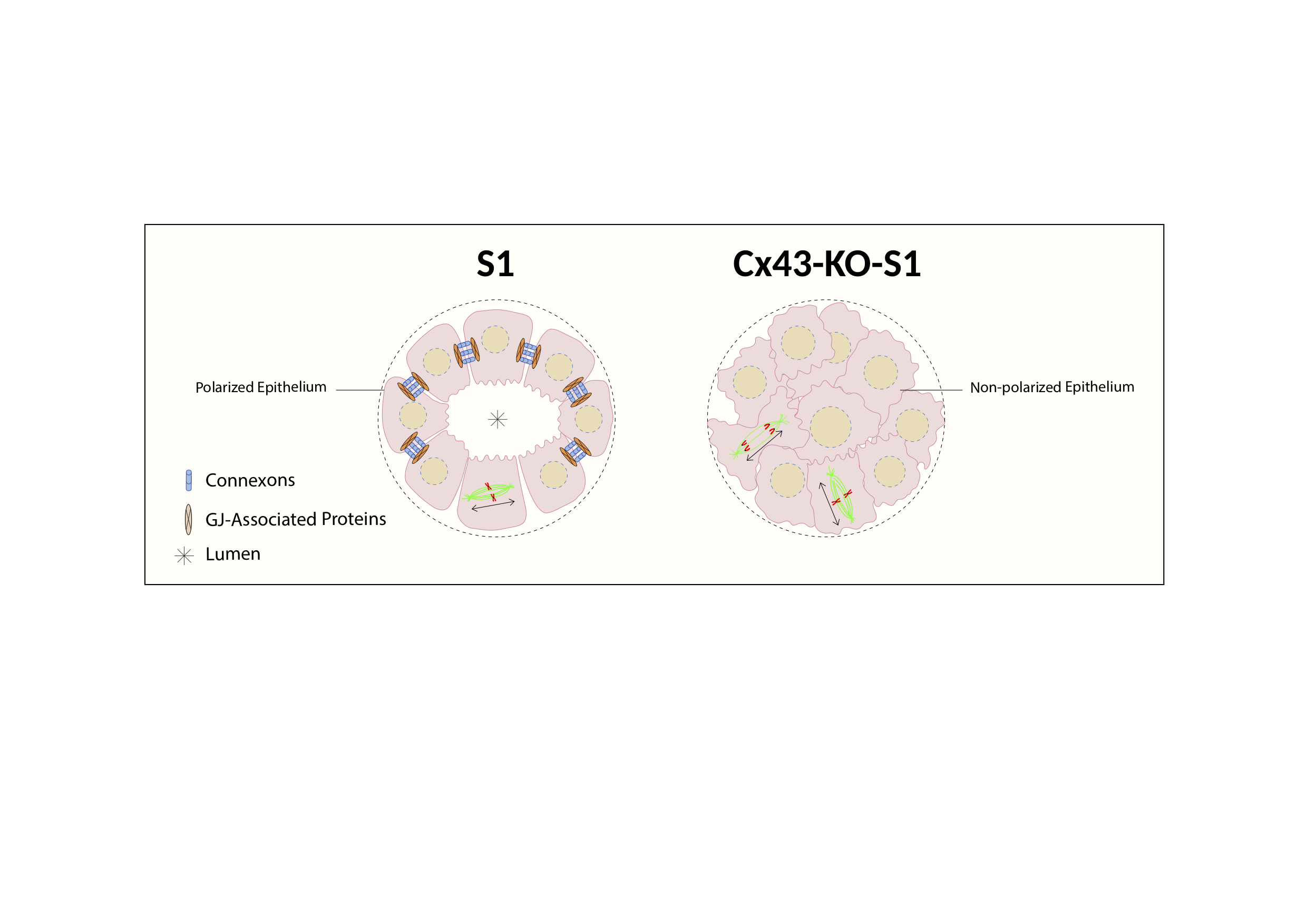
) to maintain a monolayered epithelium, in contrast to cell multilayering (
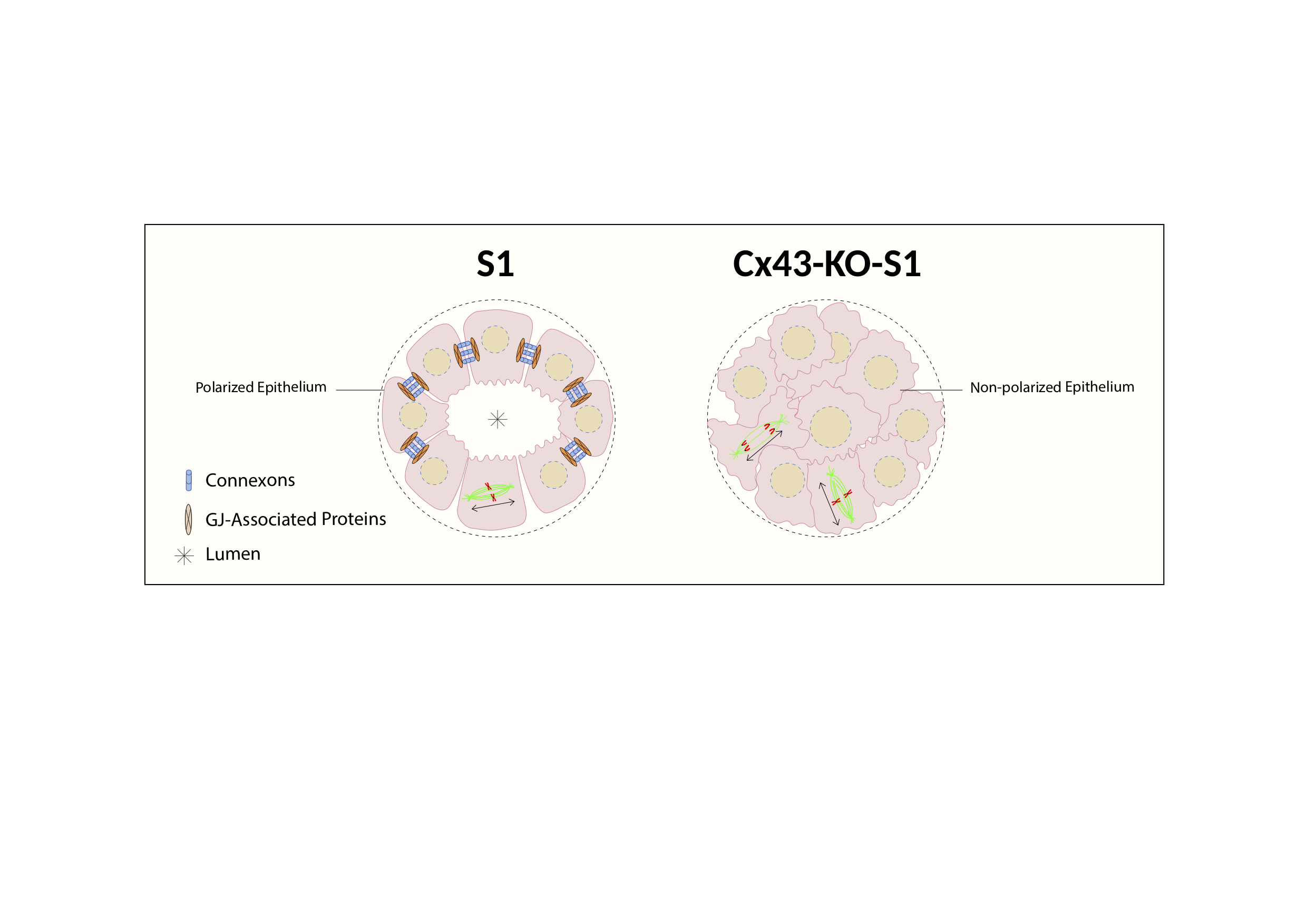
) in Cx43-KO-S1 cells *(modified from Naser Al Deen et al., 2019* [8]*)*.

**miR-182**

**Probability**


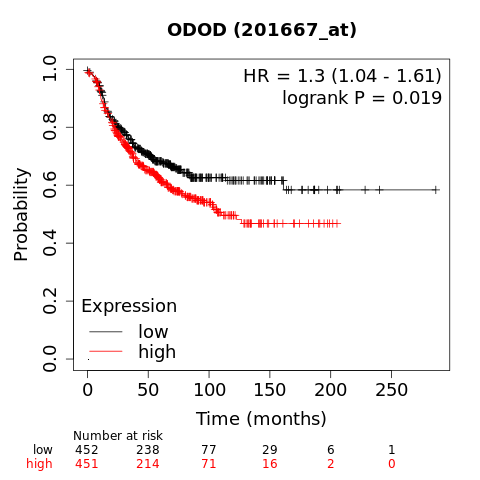


**Cx43**

**Probability**

**(a)**

**(b)**

**Supplementary Figure 2. Down-regulation of miR-182 and up-regulation of Cx43 seem to associate with poor prognosis in patients with Grade III breast tumors.** Using (**a**) METABRIC breast cancer miRNA dataset in the Kaplan-Meier Plotter [50], the survival analysis for miR-182 in 395 patients with grade III breast tumors was plotted. Using (**b**) all breast cancer mRNA datasets in the Kaplan-Meier Plotter [50, 51], the survival analysis for Cx43 in 903 patients with grade III breast tumors was plotted. *The same was performed for Grade II breast tumors and presented in (Fig. 5c & d), where up-regulation of miR-182 and down-regulation of Cx43 seem to associate with poor prognosis in Grade II breast tumors.*
